# Healthcare Seeking and Access to Care for Pneumonia, Sepsis, Meningitis, and Malaria in Rural Gambia

**DOI:** 10.4269/ajtmh.21-0362

**Published:** 2021-12-06

**Authors:** Ilias Hossain, Philip Hill, Christian Bottomley, Momodou Jasseh, Kalifa Bojang, Markieu Kaira, Alhagie Sankareh, Golam Sarwar, Brian Greenwood, Stephen Howie, Grant Mackenzie

**Affiliations:** ^1^ Medical Research Council Unit The Gambia at the London School of Hygiene & Tropical Medicine, Fajara, Kombo, The Gambia;; ^2^ Centre for International Health, University of Otago, Dunedin, New Zealand;; ^3^ London School of Hygiene & Tropical Medicine, London, United Kingdom;; ^4^ Medicines Control Agency, Kairaba Avenue, Kombo, The Gambia;; ^5^ Regional Health Team, Upper River Region, Basse, URR, The Gambia;; ^6^ Department of Paediatrics: Child and Youth Health, University of Auckland, Auckland, New Zealand;; ^7^ Murdoch Childrens Research Institute, Parkville, Melbourne, Victoria, Australia;; ^8^ Department of Paediatrics, University of Melbourne, Melbourne, Victoria, Australia

## Abstract

Children with acute infectious diseases may not present to health facilities, particularly in low-income countries. We investigated healthcare seeking using a cross-sectional community survey, health facility-based exit interviews, and interviews with customers of private pharmacies in 2014 in Upper River Region (URR) The Gambia, within the Basse Health & Demographic Surveillance System. We estimated access to care using surveillance data from 2008 to 2017 calculating disease incidence versus distance to the nearest health facility. In the facility-based survey, children and adult patients sought care initially at a pharmacy (27.9% and 16.7% respectively), from a relative (23.1% and 28.6%), at a local shop or market (13.5% and 16.7%), and on less than 5% of occasions with a community-based health worker, private clinic, or traditional healer. In the community survey, recent symptoms of pneumonia or sepsis (15% and 1.5%) or malaria (10% and 4.6%) were common in children and adults. Rates of reported healthcare-seeking were high with families of children favoring health facilities and adults favoring pharmacies. In the pharmacy survey, 47.2% of children and 30.4% of adults had sought care from health facilities before visiting the pharmacy. Incidence of childhood disease declined with increasing distance of the household from the nearest health facility with access to care ratios of 0.75 for outpatient pneumonia, 0.82 for hospitalized pneumonia, 0.87 for bacterial sepsis, and 0.92 for bacterial meningitis. In rural Gambia, patients frequently seek initial care at pharmacies and informal drug-sellers rather than community-based health workers. Surveillance underestimates disease incidence by 8–25%.

## INTRODUCTION

Pneumonia, sepsis, meningitis, and malaria are common conditions in low-income countries.^1^^,^^2^ Optimal surveillance and measurement of the burden of these conditions should take into account healthcare seeking and utilization in a given population. For example, cases of pneumonia may occur outside the healthcare delivery system, particularly in low-income countries.^3^^,^^4^ Understanding the common pathways that are used to seek care will also inform the issue of delayed care-seeking. To understand the pathways of care-seeking for common infectious diseases such as pneumonia, sepsis, meningitis, and malaria, it is necessary to consider issues related to the household, the drug retailer, and also the health facility. Most surveys were conducted at the household level, and investigators have found the percentage of children with symptoms suggestive of pneumonia that present to health facilities in Africa ranges from 23% to 85%.^5^^–^^7^ In Ghana, 56% of suspected meningitis cases were taken first to a clinic and 29% to a hospital.^8^ Care seeking for symptoms of malaria in Africa is influenced by the age and education of caregivers, knowledge of the condition, the presence of community care programs, and availability of drug therapy at the community or facility level.^9^^–^^11^

Drug retailers are generally closer to home than formal health facilities and are widely used sources of drugs for febrile illness in sub-Saharan Africa.^12^^,^^13^ Moreover, their service is faster, and their weekly opening hours may be twice as long as those of government health facilities.^14^ As drug stock-outs are common in public facilities, medicine retailers form an important alternative supply, and their staff are often perceived as being more friendly and approachable.^15^ In fee-for-service health systems, cost is often an important motivation for using medicine retailers.^16^ Even when inexpensive or free drugs are available, patients may use retailers to avoid the travel and time costs involved in accessing formal care.^16^ Religion, education, and gender also play a large role in healthcare-seeking behavior in Africa.^17^ In rural Gambia, patient knowledge of dispensed drugs is very poor and this has implications for treatment compliance, outcome, and cost.^18^

There are few data from Africa describing population-based estimates of the proportion of individuals who attend health facilities when symptoms of pneumonia, sepsis, meningitis, or malaria develop.^19^^–^^21^ In rural settings, physical distance to a health facility is a known determinant of healthcare utilization.^22^^–^^24^ One comprehensive study found that 38% of childhood pneumonia cases in Africa do not reach health facilities.^4^ In Nigeria, common barriers to sepsis care in children are lack of parental awareness of early signs, poor access to services, parental education, lack of medical equipment, and a lack of consensus for sepsis management.^25^

The objectives of this study were to: 1) describe the initial sources of healthcare in the community sought by patients with symptoms of pneumonia, sepsis, meningitis, or malaria; 2) estimate the proportion of patients that sought care elsewhere before presenting to a health facility; 3) identify pathways of healthcare seeking; and 4) describe the relationship between healthcare utilization and distance to a health facility, and so estimate the sensitivity of facility-based surveillance.

## MATERIALS AND METHODS

Data to address the study objectives were collected through a cross-sectional household survey, facility-based exit interviews, interviews with staff and customers of private pharmacies, and during population-based surveillance for pneumonia, sepsis, and meningitis.

### Setting.

Surveys were conducted between August 2013 and March 2014 and surveillance was conducted between June 2008 and December 2017 in the part of Upper River Region (URR) of The Gambia covered by the Basse Health and Demographic Surveillance System (BHDSS).^19^^–^^21^ In 2013, the population of the BHDSS was estimated to be 175,069. Approximately 19% of the population were aged 2 to 59 months, 29% were aged 5 to 14 years, and 52% were aged 15 years and above. The Gambian public health delivery system comprises referral hospitals, primary and secondary care facilities, and village-based services. The major health center in URR is Basse Health Center (BHC), which is both a primary and secondary care facility, and the facility has now been upgraded to a district hospital. Eight peripheral clinics also provide inpatient and outpatient care and may refer patients to BHC. In The Gambia, treatment at government health facilities is free of charge for children below 14 years. Surveillance for pneumonia, sepsis, meningitis, and malaria was conducted among patients aged ≥ 2 months resident in the BHDSS. Rapid diagnostic tests (RDT) for malaria were routinely used at all facilities^19^^–^^21^ Clinicians used standardized surveillance criteria (Table 1) to identify patients with suspected pneumonia, sepsis, meningitis, or combinations of the three. Standardized investigations were performed based on surveillance diagnosis and treatment was provided based on national guidelines.

**Table 1:** The criteria for suspected pneumonia, sepsis, meningitis, or malaria used for the community household survey^19^

Suspected syndrome	Case definition	
	≥ 2 months and < 5 years	≥ 5 years
Pneumonia	Lower chest wall indrawing, AND fast breathing with either cough or difficulty breathing	Lower chest wall indrawing, AND cough
Sepsis	Fever, AND either not eating, unable to sit, irritable, or no energy	Fever, AND either coma or unable to sit
Meningitis	Fever, AND either stiff neck, convulsion, or coma	Fever, AND either stiff neck or photophobia
Malaria	Fever, AND either vomiting, convulsion, or shaking	Fever, AND either shaking or convulsions

Symptoms are self-reported by the individual or parent of the child.

Malaria is endemic in the study region with peak transmission occurring during rainy season months of August to December. Diphtheria, pertussis, and tetanus immunization coverage is high (93.2%).^26^ The distance from the farthest village to a health facility is 25 km.^27^ The majority of the adult population belongs to one of the three major ethnic groups (Fula, Serahule, and Mandinka) and the majority have religious (Koranic) education only.

### Community household survey.

We conducted a household survey to measure the proportion of participants who had experienced symptoms of pneumonia, sepsis, meningitis, or malaria in the previous 3 months and to determine what actions were taken in response to these illnesses. The survey was conducted during the malaria transmission season.

**Case definitions.** In preparation for the community survey, we analyzed surveillance data from known cases of meningitis, sepsis, radiological pneumonia, or malaria carried out in URR to determine the most common symptoms associated with suspected pneumonia, sepsis, meningitis, or malaria (Table 1).^19^^–^^21^ The definitions were not mutually exclusive so there were some individuals who fulfilled the criteria for more than one condition.

**Figure 1.**
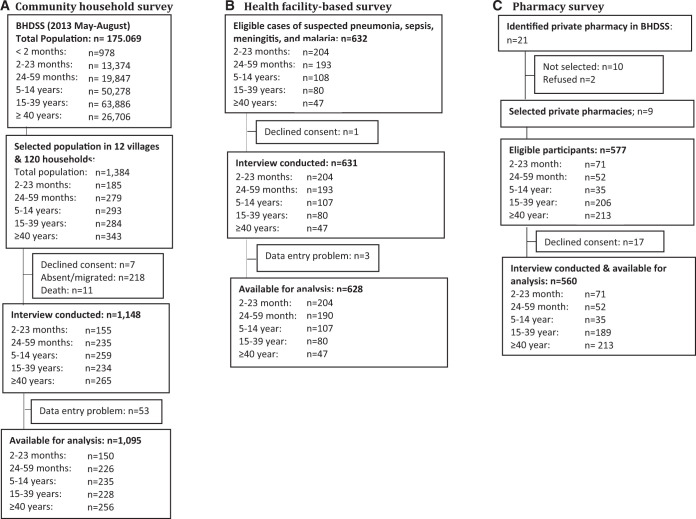
Profile of participant enrolment in the three surveys of healthcare seeking. (**A**) Community household survey. (**B**) Health facility-based survey. (**C**) Pharmacy survey.

**Sampling.** We randomly selected 12 of 225 villages within the BHDSS with probability proportional to population size, and then randomly selected 10 households per village with probability proportional to population size. Within the sampled households we selected all household members aged 2–59 months, one in three of those aged 5–14 years, one in five of those aged 15–39 years, and all those aged 40 years and above (Figure 1A). If a selected individual or household could not be enrolled after two visits, a replacement was selected.

**Figure 2.**
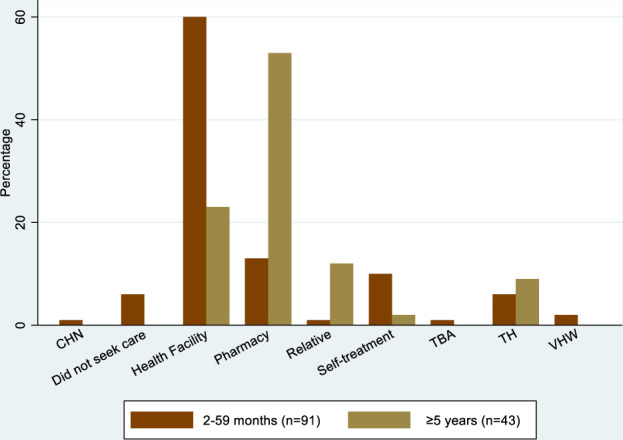
Proportion of individuals initially seeking care for their illness with different care providers; data from a community household survey. CHN = community health nurse; TBA = traditional birth attendant; TH = traditional healer; VHW = village health worker. This figure appears in color at www.ajtmh.org.

**Figure 3.**
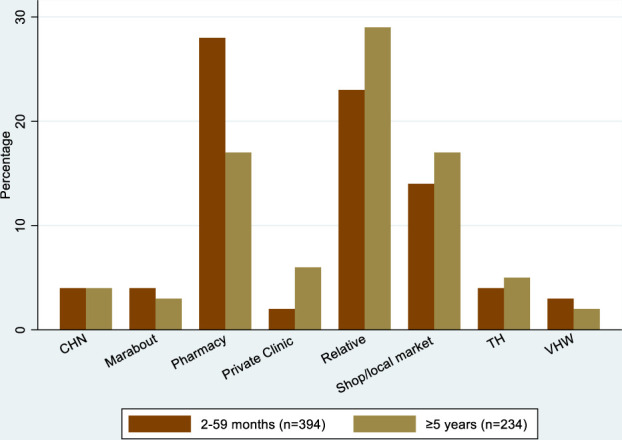
Proportion of individuals initially seeking care with different care providers before visiting the health facility; data from a facility-based survey. CHN = community health nurse; TBA = traditional birth attendant; TH = traditional healer; VHW = village health worker. This figure appears in color at www.ajtmh.org.

### Health facility-based survey.

We conducted facility-based exit interviews with patients at BHC who had been identified by surveillance staff as suspected cases of pneumonia, sepsis, meningitis, or malaria. Consecutively presenting patients with each illness syndrome in each age strata were approached for an interview at discharge from the outpatient or inpatient facility. Information was sought describing the steps taken by the patient to seek care and medical interventions that occurred on each of the days before presentation at the health facility.

### Pharmacy survey.

We conducted retail pharmacy-based interviews to document the number of patients seeking medication for febrile or respiratory illnesses, as well as their perception of the illness, the pharmacists’ (untrained) perception of the illness, and the medication purchased. Twenty-one private pharmacies that stock antibiotics were identified in the BHDSS. We mapped their location and calculated the size of the population each pharmacy was likely to serve. Nine private pharmacies serving the largest populations were selected and approached for involvement in the survey (Figure 1C). Information was sought from patients attending the pharmacies regarding the type of illness, their symptoms, whether they fulfilled the screening criteria used by the surveillance project for suspected pneumonia, sepsis, or meningitis, whether they had attended a health facility for their illness, and the nature of the medication prescribed and purchased.^19^^–^^21^

### Population-based disease surveillance.

Nurses assessed all outpatients and inpatients at all health facilities in the BHDSS using standardized criteria for referral to clinicians in Basse. For referred patients, clinicians then applied standardized criteria to identify patients with suspected pneumonia, sepsis, or meningitis and requested blood culture, lumbar puncture, and/or chest radiography. Specimens were processed in Basse using standard methods.^19^

### Data management.

Structured questionnaires for each of the surveys were administered using an electronic data collection system.

### Statistical analysis.

Age-specific proportions were estimated (2–59 months, 5–14 years, and 15 + years) with 95% confidence intervals. For the community survey and pharmacy survey, the confidence intervals were adjusted for village-level clustering. To quantify access to care, we estimated the relative decline in disease incidence with increasing distance from a health facility using five distance strata. Overall access to care in the BHDSS for each clinical category was a population-weighted sum of the stratum level ratios.^22^ Analyses were done using Stata version 15.1.

### **Ethics a** **nd consent.**

Ethics approval was obtained from the Gambia Government/Medical Research Council Joint Ethics Committee; SCC/EC 1077 and 1341. Written informed consent was obtained from all individuals aged 15 years and greater, and for younger individuals, it was obtained from their parent or legal caregiver.

## RESULTS

### Summary of recruitment.

We enrolled 1,095 individuals in the community survey, 632 in the health facility-based survey, and 560 in the pharmacy survey (Figure 1). During the surveillance period, 21,552 children aged 2–59 months were identified with suspected pneumonia, sepsis, or meningitis.

### Community household survey.

Among 376 children aged 2–59 months 6% had symptoms meeting the definition of pneumonia in the previous 3 months, 9% had symptoms of sepsis, 1% symptoms of meningitis, and 10% symptoms of malaria (Table 2). Reports of the illness were less common in older age groups. Among individuals with symptoms of pneumonia, sepsis, meningitis, or malaria, care was reportedly initially sought at a health facility in 60% (55/91) of those aged 2–59 months, 47% (8/17) in those aged 5–14 years, and 8% (2/26) in those aged ≥ 15 years; care was initially sought at a pharmacy in 13% (12/91) of 2–59 months old, 35% (6/17) of 5–14 years old and 65% (17/26) of ≥ 15 years old (Table 3, Figure 2). Care was also sought from traditional healers (7%, [9/134]), relatives (4%, [6/134]), community-based health workers (3% [4/134]), and other providers. Care outside the home was not sought for only 5% (5/91) of children aged 2–59 months. Reasons for choosing an alternative source of initial treatment to a health facility were: a better explanation of their disease would be provided (57%), advised by relatives not to attend a health facility (50%), the distance of residence from a health facility (49%), anticipation of better treatment (34%), medicines more likely to be available (28%) and more affordable (22%). Most individuals did attend a health facility at some stage during their illness although for those aged ≥ 5 years most attended three or more days after symptom onset (Table 3).

**Table 2:** Suspected syndromes reported in the previous 3 months in the community household survey

Syndrome	2–59 months (*N* = 376)		5–14 years (*N* = 235)		≥ 15 years (*N* = 484)	
	n (%)	Percentage 95% CI	n (%)	Percentage 95% CI	n (%)	Percentage 95% CI
Pneumonia	24 (6.4)	4.4, 9.2	3 (1.3)	0.4, 4.1	3 (0.6)	0.2, 2.5
Sepsis	33 (8.8)	3.6, 19.9	3 (1.3)	0.4, 4.2	2 (0.4)	0.1, 3.2
Meningitis	5 (1.3)	0.4, 4.0	2 (0.9)	0.2, 4.1	1 (0.2)	0.0, 1.6
Malaria	38 (10.1)	5.7, 17.3	11 (4.7)	2.1, 10.0	22 (4.5)	1.3, 14.2
Any of the above	91 (24.2)	14.9, 36.8	17 (7.2)	3.1, 15.9	26 (5.4)	1.6, 16.5

**Table 3:** Care seeking behavior among individuals with suspected syndromes of pneumonia, sepsis, meningitis, or malaria in the community household survey

		2–59 months (*N* = 91)		5–14 years (*N* = 17)		≥ 15 years (*N* = 26)	
		n (%)	95% CI	n (%)	95% CI	n (%)	95% CI
Where care was initially sought?	Health facility	55 (60)	46.74	8 (47)	18.78	2 (8)	1,36
	Pharmacy	12 (13)	8.20	6 (35)	11.71	65 (17)	46,81
	Community health nurse	1 (1)	0.8	0	NA	0	NA
	Village health worker	2 (2)	1.7	0	NA	0	NA
	Traditional birth attendant	1 (1)	0.9	0	NA	0	NA
	Traditional healer	5 (6)	2.18	2 (12)	3.40	8 (2)	1.36
	Relative	1 (1)	0.11	1 (6)	1.40	15 (4)	4.43
	Other	9 (10)	2.37	0	NA	4 (4)	1.20
	Did not seek care	5 (6)	2.12	0	NA	0	NA
Health facility attendance during illness	Yes	86 (95)	87.98	16 (94)	55.100	26 (100)	NA
Health facility attendance pneumonia	Yes	24 (100)	NA	2 (67)	0.100	3 (100)	NA
Health facility attendance sepsis	Yes	31 (94)	84.9 8	3 (100)	NA	2 (100)	NA
Health facility attendance meningitis	Yes	5 (100)	NA	2 (100)	NA	1 (100)	NA
Health facility attendance malaria	Yes	35 (92)	74.98	11 (100)	NA	22 (100)	NA
Number of days of symptoms before care seeking at health facility	0 days	4 (4)	1.17	1 (6)	0.59	0	NA
	1 day	23 (25)	16.38	4 (24)	8.53	3 (12)	2.48
	2 days	41 (45)	33.58	5 (29)	3.85	7 (27)	13.48
	3+ days	18 (20)	11.33	7 (41)	15.74	16 (62)	29.86
	Did not seek care	5 (6)	2.12	0	NA	0	NA

### Health facility-based survey.

The common suspected surveillance diagnoses among 394 participants aged 2–59 months were sepsis (28%), malaria (28%), and pneumonia (28%). Among 234 participants aged ≥ 5 years, the common diagnoses were malaria (57%) and pneumonia (32%). The most common initial avenue for care among the facility survey participants was retail pharmacies, 27.9% (110/394) in those aged 2–59 months and 16.7% (39/234) in those aged ≥ 5 years (Figure 3). The next most common avenue for initial care was to seek advice from a relative, 23.1% (91/394) for young children and 28.6% (67/234) for older patients. Patients sought initial care from shop keepers selling medicines and the local market [14.2% (89/628)], purchased drugs from pharmacies in 13.5% (53/394) for young children and 16.7% (39/234) for older patients respectively, before visiting the health facility.

### Pharmacy survey.

Five hundred and eight of 560 customers, (91%) sought medication for febrile illnesses. Among participants aged < 5 years the suspected diagnoses were pneumonia, sepsis, or meningitis in 81.3% (100/123). The suspected diagnoses in those aged ≥ 5 years were pneumonia, sepsis, or meningitis in 51% (224/437). Attendance at a health facility prior to attending the pharmacy was more common in children aged < 5 years than in the 5–14 years or adult age group (47%, 37%, and 30% respectively). These findings often represented the fact that although the pharmacy attendees had initially presented, and been diagnosed, at a health facility they needed to attend a private pharmacy as the recommended medicines were not available at the health facility. Children aged < 5 years attending a pharmacy were more likely to receive antibiotics (93.5% [115/123]) compared with those aged ≥ 5 years (67% [291/437]). Co-trimoxazole and amoxicillin were the most commonly dispensed medicines in all age groups, consistent with national guidelines.

### Access to care.

**Figure 4.**
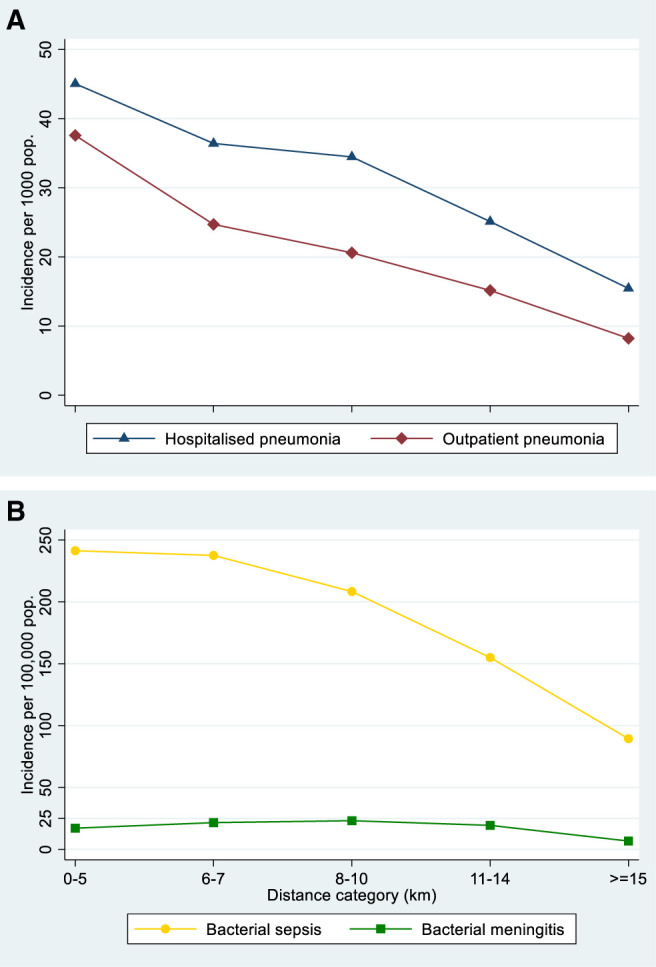
Incidence of (**A**) outpatient and hospitalized pneumonia and (**B**) bacterial sepsis and bacterial meningitis, by distance to the nearest health facility. This figure appears in color at www.ajtmh.org.

The surveillance study enrolled 21,552 children aged 2–59 months with suspected pneumonia, sepsis, or meningitis. The incidence of outpatient pneumonia, hospitalized pneumonia, and bacterial sepsis was greatest in households located < 5 km from the nearest health facility and incidence decreased with increasing distance from the health facility (Table 4, Figure 4). The incidence of bacterial meningitis was relatively constant in all distance strata 0–14 km from the nearest health facility and then fell in household ≥ 15 km from the nearest health facility (Table 4, Figure 4). Access to care ratios compared the incidence of disease in households at increasing distances away from the nearest health facility to households 0–5 km away (0–14 km away for bacterial meningitis) and were greater for bacterial meningitis than pneumonia (Table 4). Overall access to care ratios in the BHDSS, calculated as weighted averages of the stratum-specific ratios, ranged between 0.75 for outpatient pneumonia and 0.92 for bacterial meningitis (Table 4).

**Table 4:** Disease incidence and access to care ratios for disease categories by distance to the nearest health facility and for the BHDSS population overall

Distance of household to nearest health facility (km)	Disease category								
	Outpatient pneumonia		Hospitalized pneumonia		Bacterial sepsis			Bacterial meningitis	
	Incidence/1,000 pop.	Access to care ratio	Incidence/1,000 pop.)	Access to care ratio	Incidence/100,000 pop.	Access to care ratio	Incidence/100,000 pop.		Access to care ratio
0–5	37.6	1	45.1	1	241.3	1	17.1		1
6–7	24.7	0.66	36.4	0.81	237.5	0.98	21.6		1
8–10	20.6	0.55	34.5	0.76	208.3	0.86	23.1		1
11–14	15.2	0.40	25.1	0.56	155.0	0.64	19.4		1
≥ 15	8.2	0.22	15.4	0.34	89.4	0.37	6.7		0.39
Overall		0.75		0.82		0.87			0.92

BHDSS = Basse Health and Demographic Surveillance System. Incidence of bacterial meningitis was relatively constant in the distance categories 0–5 km, 6–7 km, 8–10 km, and 11–14 km and so these strata were combined to form the reference stratum for the calculation of overall access to care.

## DISCUSSION

In this study, we found that a history of symptoms of pneumonia or sepsis (15%) or malaria (10%) were common in young children during the 3 months period before the start of the survey. Care seeking commonly involved health facilities at an early stage, although in all age groups those who did attend health facilities frequently sought care from a wide range of other providers. Formal and informal retailers of medicines formed an important part of care-seeking for many individuals, both before and after presentation to health facilities. The decrease in disease incidence with increasing distance between households and health facilities suggests that access to care decreases with increasing distance away from health facilities.

Our findings are consistent with a WHO report in 2013 that 68% of Gambian children with symptoms of pneumonia were taken to a healthcare provider.^6^ We estimated that 75% of children with outpatient pneumonia presented to a health facility. Similarly, our findings are consistent with a Gambian report in 2008–2012 in which 69% of children suspected to have pneumonia sought appropriate medical care.^28^ In Tanzania, 77.2% of adult patients had sought care elsewhere before presentation at a health facility for the same illness episode.^29^ In sub-Saharan Africa, where most pneumonia deaths occur, United Nations Children’s Fund (UNICEF) reports indicate that only 57% of children with symptoms of pneumonia are taken to a source of care with little progress in care-seeking behavior since 2000.

In the community, young children, and to a lesser extent children aged 5–14 years, were much more likely than adults to initially seek care at a health facility. These findings are consistent with most community-based surveys in Africa.^29^^–^^31^ Investigations into access to healthcare often fail to embrace all the proposed components of access, evaluating only traditional factors such as distance, travel time, cost of care, maternal education, and household income and wealth.^32^^–^^37^ Other factors are also felt to be important. It has been suggested that families are denied access to healthcare because of lack of knowledge of services available, shortage of money, and absence of a village health worker at critical periods, which is consistent with our findings.^38^ Van den Broeck et al.^39^ found that a distance of over 5 km from a healthcare center was positively associated with childhood mortality among those living in a rural African community, while Armstrong Schellenberg et al.,^36^ Sankoh et al.,^40^ and Mtango et al.^41^ using either time or distance found no difference between cases (child deaths) and controls (children living at the time of case death) relative to the time or distance of residence from a health facility.

Similar to other reports,^42^ the use of pharmacies in our setting was related to a lack of medication in Government facilities as well as being a “first port of call” for adults. We found that ease of access to a pharmacy was important in care-seeking. In the Democratic Republic of Congo, 16–18% of children were initially taken to a private pharmacy.^5^ A study in Nigeria reported that residence close to a pharmacy was one of the main motivations for preferring pharmacies as a primary source of healthcare.^43^ A recent study in Ghana indicated that the majority of adult pharmacy users believe that community pharmacies can handle minor ailments.^44^ In 2013, the World Bank reported that about 80% of Africans rely on public health facilities but those facilities suffer from chronic shortages of critical drugs.^45^ The common initial use of pharmacies by adults in our setting is strongly related to substantial user-fees and a lack of medicines at health facilities.^46^ Substantial barriers must be overcome if the aspiration of universal healthcare, with reliable supply chains, is to be achieved.^45^ Several reports have suggested private medicine retailers are fast becoming key players in promoting access to medicines in low- and middle-income countries.^15^^,^^47^

Our data indicate underutilization of appropriate providers in the community (community health nurses and village health workers) with only 7% of children seeking such care before presenting at a health facility, while 41% sought prior care from a retailer of medicines. This finding is consistent with studies from other countries showing the importance of accessibility in healthcare-seeking behavior.^43^^,^^48^

The majority of patients preferred starting with self-treatment at home. Studies elsewhere in Africa have reported similar findings.^49^^,^^50^ Caregivers preferring home-based treatment often obtained advice from neighbors or relatives or sought traditional treatment. Decisions to seek care outside the home in our, and other African settings, were influenced by perceptions that a provider would explain the disease, advice not to attend a health facility, and close proximity to a pharmacy or traditional healer.^51^^–^^53^ Other facilities were chosen because they were perceived to be of better quality. These observations suggest that along with accessibility, the perceived quality and effectiveness of a health service are also important factors that influence healthcare-seeking.^53^

We found that 25%, 45%, and 20% of children were brought to care at a health facility on day 1, 2, and 3 respectively, after the appearance of symptoms. Despite the majority of parents or primary caregivers having very low educational backgrounds and significant knowledge gaps in the perception of illness and its management,^27^ substantially delayed care-seeking is not common. Patient knowledge of their dispensed medication is very poor in our setting and can result in medication errors.^18^ In our study 11.4% and 13% of under-five children sought care and purchased both antibiotics and antimalarial drugs and only antimalarial drugs respectively from the pharmacy during their illnesses. Several studies have found that delays in seeking appropriate care are one of the major risk factors for fatal pneumonia.^54^^,^^55^ Our finding that 44% of carers of febrile young children sought care first from a pharmacy is consistent with several Ugandan studies.^56^^,^^57^ Overall access to care in our settings appears better than reported in Nigeria (access to care distance decay of 9% per km) and coastal Kenya (45% of pneumonia cases and 30% of meningitis cases missed by hospital surveillance).^22^^,^^24^^,^^58^

The strengths of our study are the inclusion of surveys at the household, pharmacy, and facility level and high response rates. A potential limitation of the community survey was recall bias from retrospective reporting and respondent desire to provide an expected response. Observer bias may have occurred when interviewers collected data on symptoms in the facility-based survey and these data may also be biased by respondent desire to provide an expected response. Some symptoms were reported by the parents and due to their complexity these reports may not be reliable. Our analysis of access to care using “distance decay” in observed disease incidence may have omitted other factors influencing access to care, such as travel time, mode of travel, and maternal education. However, the overall access to care ratios should provide reasonable estimates of surveillance sensitivity. The findings may not reflect the situation during the COVID-19 pandemic; however, our anecdotal experience is that following an initial disruption due to COVID19, healthcare-seeking patterns have now returned to normal.

In conclusion, we found that the majority of children and adults in the BHDSS initially seek care and drugs outside the formal healthcare system and lack access to community health nurses or village health workers. These data suggest there is scope to improve access to health services in rural Gambia, particularly the availability and dispensing of drugs. Our findings indicate that the sensitivity of our surveillance is relatively good with a limited proportion of pneumonia, sepsis, and meningitis cases being missed. A mixed method formative evaluation could be repeated now in 2021/2 to review any changes—and then assist in the design of recovering health services. The results of this study may assist government planning and implementation of community and facility services. Likewise, our findings can inform policy to better structure the provision of healthcare through the private system to align with community preferences, reform the regulation of private pharmacies and drugs.
